# Effect of Region-of-Interest Prompting on Gemini 2.5 Pro in MRI Classification of Anterior Cruciate Ligament Injury

**DOI:** 10.7759/cureus.102850

**Published:** 2026-02-02

**Authors:** Nitin Chetla, Shivam Patel, Luis Rodriguez, Harlene Kaur, Andrew Bouras, William Wang, Adamya Gupta, Samuel Rice

**Affiliations:** 1 Medicine, University of Virginia School of Medicine, Charlottesville, USA; 2 Data Science, University of Virginia, Charlottesville, USA; 3 Medicine, Johns Hopkins University School of Medicine, Baltimore, USA; 4 Allopathic Medicine, University of Massachusetts Chan Medical School, North Worcester, USA; 5 Osteopathic Medicine, Nova Southeastern University Dr. Kiran C. Patel College of Osteopathic Medicine, Clearwater, USA; 6 Allopathic Medicine, University of Southern California Keck School of Medicine, Los Angeles, USA; 7 Orthopedics and Physical Rehabilitation, University of Massachusetts Chan Medical School, North Worcester, USA

**Keywords:** anterior cruciate ligament, artificial intelligence, diagnostic imaging, knee mri, large language model, machine learning, musculoskeletal imaging, prompt engineering, region of interest, sports injury

## Abstract

Background: Artificial intelligence (AI) has shown promise in musculoskeletal imaging, yet the diagnostic contribution of large language models (LLMs) remains unclear. Prompt engineering may critically shape performance.

Objective: To evaluate the diagnostic accuracy of Google Gemini 2.5 Pro in classifying anterior cruciate ligament (ACL) status on knee magnetic resonance imaging (MRI) and to compare three prompting strategies; the primary endpoint was weighted F1-score.

Methods: A retrospective diagnostic study used 150 proton-density fat-suppressed (PD-FS) knee MRI volumes (50 each: healthy, partially injured, completely ruptured) drawn from a publicly available dataset (Clinical Hospital Centre Rijeka, Croatia; 2006-2014). Gemini 2.5 Pro received multimodal inputs via the official Python software development kit (SDK). Three prompts were tested: (P1) general series prompt, (P2) technical-description prompt, and (P3) region-of-interest (ROI)-focused prompt. Outputs (A = healthy, B = partial, C = ruptured) were compared with radiologist labels. Accuracy, precision, recall, specificity, F1 score, confusion matrices, and mean inference time were computed (scikit-learn v1.5.0). Ethical approval was waived because the data were de-identified and publicly available.

Results: Mean inference time was 2.1 ± 0.3 seconds per volume. ROI prompting (P3) yielded the highest weighted F1-score (0.31), while macro recall (0.35) and macro specificity (0.67) were similar across prompts. Confusion matrices showed improved discrimination of completely ruptured ACLs with P3.

Conclusions: Despite a minor improvement in the weighted F1-score with Prompt 3, all prompts demonstrate poor overall classification performance, with low sensitivity and accuracy. The consistently overlapping confidence intervals indicate that prompt variations alone are insufficient to meaningfully enhance model performance. These findings suggest fundamental limitations in the model's ability to handle this task rather than suboptimal prompting.

## Introduction

Anterior cruciate ligament (ACL) injuries represent one of the most common and debilitating musculoskeletal conditions, accounting for up to half of all knee injuries [1]. Diagnosis typically combines physical examination and patient history, yet magnetic resonance imaging (MRI) remains the gold standard for confirming ligament integrity and guiding surgical decision-making [2,3]. Despite advances in imaging techniques, acute ACL ruptures are frequently misdiagnosed as uncomplicated knee sprains, underscoring the need for improved diagnostic precision [4]. Accurate classification of ACL status is essential to prevent secondary injury, support appropriate surgical intervention, and inform postoperative rehabilitation [5].

Artificial intelligence (AI) has shown increasing promise in musculoskeletal radiology, particularly through the use of deep learning models for pattern recognition and automated diagnosis [6,7]. However, the diagnostic role of large language models (LLMs) in interpreting imaging data remains largely unexplored. LLMs such as Google's Gemini 2.5 Pro possess multimodal capabilities that allow integration of visual and textual inputs, offering potential for streamlined interpretation of medical images [8]. The effect of prompt engineering, the structured phrasing of model inputs, on diagnostic accuracy is of growing interest, as prompt structure may critically influence output reliability and reproducibility [9,10]. Moreover, the increasing availability of open medical datasets has enabled more practical AI-based diagnostic modeling [5,11].

This study evaluates the diagnostic accuracy of Gemini 2.5 Pro in classifying ACL integrity on knee MRI and compares three prompting strategies: general descriptive prompting, technical imaging prompting, and region-of-interest (ROI)-focused prompting. By identifying optimal prompting frameworks, this work aims to inform future applications of LLMs in musculoskeletal imaging and enhance the interpretability and clinical utility of multimodal AI systems.

This article was previously posted to the Research Square preprint server on August 1, 2025.

## Materials and methods

Study design and dataset

This retrospective diagnostic evaluation used a publicly available knee MRI dataset containing 917 de-identified 12-bit grayscale volumes acquired at the Clinical Hospital Centre Rijeka (Croatia) between 2006 and 2014 on a Siemens Avanto 1.5T scanner using a proton density weighted fat suppression (PD-FS) sequence. Volumes were labeled by radiologists as (1) healthy, (2) partially injured, or (3) completely ruptured anterior cruciate ligament (ACL).

Sampling

For this study, 150 MRI volumes were selected by random sampling with equal class balance (50 per class). The sampling procedure and random seed were fixed prior to inference and held constant across prompting strategies.

Image preprocessing and inputs

Volumetric files were decoded into 2D axial slices and exported as PNG images. Two image input formats were prepared per case: (1) full-volume image set: axial slices from the entire volume and (2) ROI image set: axial slices with a rectangular ACL-centered region of interest derived from accompanying dataset annotations. ROI slices were additionally visualized with a red bounding box to indicate the region supplied to the model.

Prompts 1 and 2 were paired with the full-volume image set, whereas Prompt 3 was paired with the ROI image set.

Region-of-interest specification

ROI slices were generated using the dataset-provided annotation/metadata to localize the ACL region within each volume. The ROI definition process was deterministic given the source annotations; inter-rater reliability was not assessed because ROI selection was not performed independently by multiple reviewers.

Model and prompting strategies

Gemini 2.5 Pro Preview (June 2025, Google) was accessed via the official Google Generative AI Python SDK. Three prompting strategies were evaluated: a general series prompt (Prompt 1), a technical-description prompt (Prompt 2), and an ROI-focused prompt (Prompt 3). The verbatim prompt templates are provided in Appendix A. Inference settings were held constant across all runs to isolate the effect of prompt design.

For each case, the model received a structured multimodal input consisting of the prompt text and the corresponding slice sequence (full-volume or ROI set as defined above). The model was instructed to return only a single-character label: A = Healthy, B = Partially Injured, and C = Completely Ruptured.

Outcomes and statistical analysis

Model predictions were logged and compared to the ground-truth class label. Performance was assessed using accuracy, precision, recall (macro-averaged), specificity (macro-averaged), weighted F1-score, and confusion matrices. Ninety-five percent confidence intervals were estimated using nonparametric bootstrap resampling (1,000 resamples). Mean inference time per MRI volume was calculated from the recorded API request-to-response duration.

Ethics

Ethical review was waived because all data were de-identified and publicly available.

Data and code availability

The analysis code, fixed sampling procedure (including random seed), and prompt templates are available from the corresponding author upon reasonable request.

## Results

Gemini 2.5 Pro achieved a mean inference time of 2.1 ± 0.3 seconds per MRI volume. Comparative performance metrics across the three prompting strategies are summarized in Table 1. Overall sensitivity and specificity were consistent across all prompting strategies. ROI-based prompting (Prompt 3) demonstrated the most balanced performance, but this difference is not statistically significant based on the reported confidence intervals.

**Table 1:** Comparative performance metrics of Gemini 2.5 Pro across three prompting strategies for ACL classification. Data are expressed as mean values with 95 percent confidence intervals, shown as x (y, z). ROI-focused prompting (Prompt 3) achieved the highest weighted F1-score and precision.

Model	Sensitivity (macro recall)	Specificity (macro specificity)	Accuracy	Weighted F1-score
Prompt 1	0.35 (0.27, 0.42)	0.67 (0.60, 0.75)	0.35 (0.27, 0.42)	0.20 (0.14, 0.27)
Prompt 2	0.34 (0.26, 0.42)	0.67 (0.59, 0.75)	0.34 (0.26, 0.42)	0.21 (0.15, 0.28)
Prompt 3	0.35 (0.27, 0.42)	0.67 (0.60, 0.75)	0.35 (0.27, 0.42)	0.31 (0.24, 0.38)

**Figure 1:**
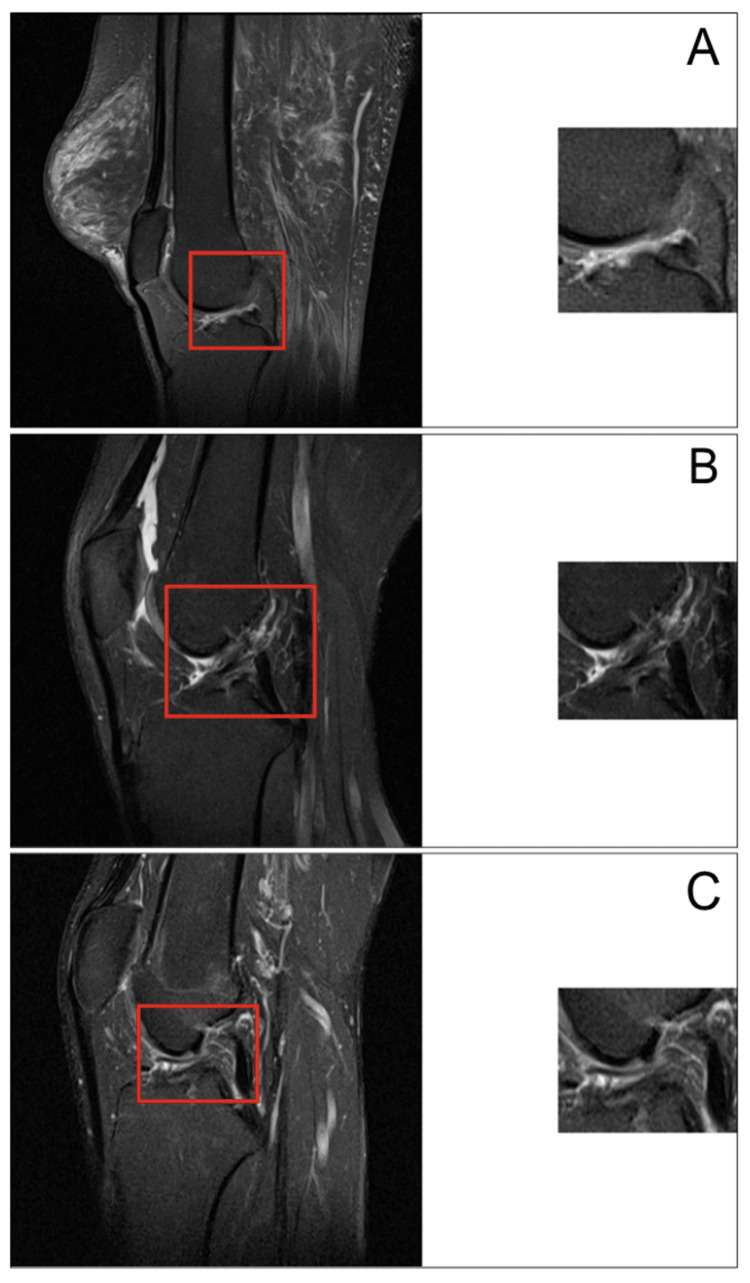
(A) Healthy knee showing intact anterior cruciate ligament (ACL) fibers. (B) Partial tear demonstrating signal heterogeneity and focal thinning. (C) Complete rupture with full discontinuity and fiber retraction. Red bounding boxes denote the manually defined regions of interest provided to the model. Representative sagittal proton density fat-suppressed knee MRIs used for model inference and evaluation, with corresponding magnified ACL ROIs on the right.

Qualitative image analysis (Figure 1) revealed that the model exhibited greater discriminative ability in recognizing disrupted ligament fibers and altered morphology in completely ruptured ACLs when supplied with ROI-focused contextual cues.

General and technical prompts performed similarly but demonstrated reduced consistency in differentiating between partial and complete ruptures. The results indicate that localized visual focus and targeted textual context enhance multimodal model interpretability for musculoskeletal imaging tasks, reinforcing the importance of structured prompt design in LLM-based diagnostic workflows.

## Discussion

The performance differences observed across prompting strategies highlight the influence of prompt engineering on LLM interpretation of medical images [12]. Among the tested configurations, the ROI-focused prompt produced the most balanced performance metrics, achieving the highest weighted F1-score and precision. This finding suggests that anatomically localized context improves the model's ability to differentiate subtle ligament abnormalities on MRI. The addition of ROI bounding boxes likely guided Gemini 2.5 Pro's visual attention toward diagnostically relevant features, such as ligament continuity, fiber orientation, and signal intensity changes.

General and technically descriptive prompts performed comparably but demonstrated lower classification consistency, particularly when distinguishing partial from complete ruptures. This variability aligns with prior research showing that general multimodal LLMs can misinterpret diffuse or context-poor visual cues [3,6]. The limited accuracy across all prompts also reflects the complexity of ACL morphology and the small sample size used in this preliminary diagnostic study.

Compared with conventional deep learning approaches, the performance of Gemini 2.5 Pro in this study was notably lower. Bien et al. [13] reported that their MRNet convolutional neural network achieved an area under the receiver operating characteristic curve (AUC) of 0.965 for ACL tear detection on knee MRI, with a sensitivity of 0.759 and a specificity of 0.968 when operating at the clinical threshold. Similarly, Chang et al. [14] demonstrated that deep learning models trained specifically for musculoskeletal imaging achieved sensitivity exceeding 0.90 for complete ACL tears. The substantial performance gap between task-specific deep learning models and general-purpose LLMs underscores that multimodal LLMs are not yet optimized for specialized radiological interpretation. This disparity likely stems from the absence of domain-specific training data and the architectural differences between vision-language models and dedicated convolutional neural networks designed for medical image analysis.

The influence of prompt engineering observed in our study is consistent with findings in other medical AI applications. Nori et al. [15] demonstrated that structured prompting significantly improved GPT-4's performance on medical licensing examinations, with chain-of-thought and few-shot prompting strategies yielding accuracy improvements of 5-10 percentage points over zero-shot approaches. In radiology-specific contexts, Bhayana et al. [16] found that GPT-4V exhibited variable performance across imaging modalities, performing better on chest radiographs than on cross-sectional imaging such as MRI, and highlighted the importance of prompt specificity in eliciting accurate interpretations. Our finding that ROI-focused prompts outperformed general prompts extends this observation to musculoskeletal imaging, suggesting that spatial localization cues may be particularly valuable for LLM-based image interpretation.

The modest overall accuracy observed in this study also reflects inherent challenges in ACL injury classification. Partial tears present diagnostic difficulty even for experienced radiologists, with reported inter-observer variability ranging from moderate to substantial agreement [17]. The three-class classification task (healthy, partial, complete) is inherently more challenging than binary tear detection, which may partially explain the lower performance metrics compared with studies employing binary classification schemes.

Despite modest sensitivity and specificity, the study provides early evidence that multimodal LLMs can process medical image data when paired with structured textual input. Future iterations of Gemini and similar architectures may achieve improved diagnostic reliability through expanded training datasets, integration with domain-specific medical vision encoders, and reinforcement learning from radiologist feedback. In practice, optimized prompt structures could serve as low-code interfaces for clinical decision support, enabling radiologists to iteratively refine model outputs without retraining underlying networks.

However, several limitations must be acknowledged. The dataset was relatively small and derived from a single institution, which limits generalizability. Manual ROI selection introduces observer bias and may not scale to larger datasets. Additionally, Gemini 2.5 Pro is a general-purpose model not fine-tuned for radiology, which likely constrained its discriminative power. Future work should evaluate reproducibility across different imaging modalities, LLM architectures, and automated ROI extraction pipelines.

Overall, these findings demonstrate that prompt structure is not merely a linguistic variable but a fundamental determinant of model interpretability and performance in multimodal AI. Incorporating anatomically grounded, context-aware prompts can enhance diagnostic precision and may represent a key step toward clinically deployable, human-aligned LLM imaging systems [12].

## Conclusions

This study demonstrates that prompt design significantly influences the diagnostic performance of Gemini 2.5 Pro in classifying ACL integrity on knee MRI. Among the prompting strategies tested, ROI-focused prompts achieved a superior weighted F1-score compared to general or technically descriptive prompts. These results suggest that anatomically targeted contextualization enhances model interpretability and diagnostic discrimination.

While overall accuracy remains limited for clinical use, the findings underscore the potential of LLMs to contribute to radiologic interpretation when combined with structured input design. Future research should expand dataset diversity, automate ROI generation, and evaluate multimodal prompt frameworks across additional musculoskeletal imaging tasks. Optimized prompting may serve as a practical bridge between general-purpose LLMs and domain-specific medical AI applications, supporting more consistent and explainable image-based decision making.

## Appendices

Appendix A: Verbatim prompt templates used for Gemini 2.5 Pro inference

Prompt 1 (General series prompt; full-volume axial slices provided):

Here is a series of MRI images. Based on the imaging features of the anterior cruciate ligament (ACL), classify the condition into one of the following categories:

A) Healthy

B) Partially injured

C) Completely ruptured

Only return the letter; for example, if you believe the classification is Healthy, then your response should be “A”.

Prompt 2 (Technical-description prompt; full-volume axial slices provided):

You are given a 12-bit grayscale knee MRI volume extracted using a proton density weighted fat suppression technique. Based on the imaging features of the anterior cruciate ligament (ACL), classify the condition into one of the following categories:

A) Healthy

B) Partially injured

C) Completely ruptured

Only return the letter; for example, if you believe the classification is Healthy, then your response should be “A”.

Prompt 3 (ROI-focused prompt; ROI slices with bounding boxes provided):

You are given a 12-bit grayscale knee MRI volume extracted using a proton density weighted fat suppression technique. A rectangular region of interest (ROI) has been manually selected from either the left or right knee. Based on the imaging features of the anterior cruciate ligament (ACL), classify the condition into one of the following categories:

A) Healthy

B) Partially injured

C) Completely ruptured

Please base your classification on signal intensity, continuity of the ligament, and any signs of disruption or abnormal morphology.

Only return the letter; for example, if you believe the classification is Healthy, then your response should be “A”.

For all prompts, the model output was constrained to a single character (A, B, or C) corresponding to the three classes.
